# Drug-Related Problems and Polypharmacy in Nursing Home Residents: A Cross-Sectional Study

**DOI:** 10.3390/ijerph19074313

**Published:** 2022-04-04

**Authors:** Raquel Díez, Raquel Cadenas, Julen Susperregui, Ana M. Sahagún, Nélida Fernández, Juan J. García, Matilde Sierra, Cristina López

**Affiliations:** 1 Pharmacology, Department of Biomedical Sciences, Faculty of Health Sciences, Institute of Biomedicine (IBIOMED), University of León, 24071 León, Spain; rdielz@unileon.es (R.D.); rcads@unileon.es (R.C.); mnferm@unileon.es (N.F.); jjgarv@unileon.es (J.J.G.); msiev@unileon.es (M.S.); clopcd@unileon.es (C.L.); 2 Applied Mathematics, Department of Mathematics, University of León, 24071 León, Spain; jsusl@unileon.es

**Keywords:** drug-drug interactions, drug-related problems, elderly, medication, nursing home, polypharmacy

## Abstract

At present, 19.2% of the Spanish population is aged 65 or older. Polypharmacy is a frequent condition among the elderly, especially in those living in nursing homes, which is associated with adverse outcomes, such as adverse drug events or drug-drug interactions. This study aimed to assess the pattern of polypharmacy in a nursing home in Leon, one of Spain’s most ageing regions, and its relationship with different drug-related problems. A descriptive, observational, and cross-sectional study design was used; 222 residents were involved in this study. Data on drug use were collected from medical charts. Information was screened with the software CheckTheMeds, BOT PLUS and Drug-Reax. Residents were on a median of 7 medicines. Polypharmacy and inappropriate medications were present in 78.8% and 96.8% of residents, respectively. Drug-related problems were present in almost all the populations evaluated. Drug-drug interactions were very common in participants (81.1%), being severe/moderate in 24.7%. A high prevalence of polypharmacy and drug-related problems in the nursing home population assessed has been observed. A significantly higher risk of suffering drug-drug interactions was revealed for increasing polypharmacy and anticholinergic risk. A regular evaluation of drug prescribing in nursing home residents is necessary to minimize drug-related problems risk.

## 1. Introduction

The world’s population is ageing, especially in the Western world, where the elderly represents an increasingly large group of the population. This trend is even more marked in Spain. In 2021, the population over 65 years in this country amounted to 9.38 million (nearly a fifth of the Spanish population), having increased by more than 2 million in 20 years [1]. Castile-Leon is one of the Spanish regions with the highest proportion of people over 65 years (25.4%) [2], and it is estimated that these values will continue to rise in the coming years. According to Eurostat’s projections, in 2050 two provinces of Castile-Leon, Zamora and Leon, will become the first and fourth regions, respectively, with the oldest population in the European Union (EU) [3].

Ageing leads to an increased burden on health care systems, as older adults often have multiple diseases and require several medications [4], which favours polypharmacy. Polypharmacy is defined as “the administration of many drugs at the same time or of an excessive number of drugs” [5]. This factor, together with comorbidity and age-related physiological changes, may explain the increased vulnerability of the elderly to potential drug-drug interactions (DDIs) and, consequently, to adverse drug events [6,7,8]. Moreover, the worsening of pre-existing conditions or the ineffectiveness of treatment in some of these patients may also help to confound all these effects.

This situation is more evident for nursing home (NH) residents, who are provided care and support for daily living activities as well as medical and non-medical assistance. Institutionalised residents tend to use more drugs and in larger doses, and they usually show other characteristics (poor mental health, frailty, or lower physical activity, among others) which exposes them to DDIs [9,10,11] and, in general, drug-related problems (DRP). Several studies have indicated that the use of potentially inappropriate medication (PIM) in the elderly is also common in NH settings: 23.7–70% of residents had at least one PIM [12,13,14,15,16], and 25.1–37.8% experienced potential DDIs, being 72% of moderate or major severity [9,11].

Although some studies have evaluated polypharmacy in Spain, to the best of our knowledge, none of them has identified the pattern of polypharmacy in an elderly population living in a nursing home, the associated factors, and its relationship with different drug-related problems (DRP), especially clinically relevant DDIs. The results obtained can be used to minimise the consequences of polypharmacy in order to try to avoid it and with it, the associated drug-related problems.

## 2. Materials and Methods

An observational, descriptive, and cross-sectional study was carried out in a NH located in the province of Leon (Spain). Information on institutionalised elderly and their treatments was collected from February to July 2021 after having reviewed medical charts. Data were recorded from the NH management software and completed with clinical information obtained from the physician of the NH. The collection was carried out by guaranteeing the identity of the patients and the confidentiality of the data. The Strengthening the Reporting of Observational Studies in Epidemiology (STROBE) Statement was used to report data [17].

The study population consisted of all residents aged 65 years or older. The medications were, in all cases, chronic treatments administered by oral, inhalation, or ophthalmic routes. All drugs included in this study required a prescription and had been administered to residents for at least 1 month prior to data collection. Over the counter (OTC) medications, nutritional supplements, and herbal medicines were not considered. According to previous studies, polypharmacy status was categorised into three groups: non-polypharmacy (0–4 medicines), polypharmacy (5–9 medicines), and excessive polypharmacy (at least 10 medicines) [18,19,20,21,22]. Medications, demographic characteristics (age and gender) and pathologies (International Classification of Diseases, Tenth Revision, ICD-10) of the NH residents were registered.

All medications were categorised according to the World Health Organization (WHO) anatomical-therapeutic-chemical (ATC) classification system [23]. Any combination medicine (multicomponent products) was considered a single medicine.

Information obtained was evaluated with the help of several specific software. Initially, the tool CheckTheMeds (CheckTheMeds v.3.6.4, CheckTheMeds Technology SL, Almeria, Spain) was used. CheckTheMeds is a program employed in hospitals to process the information of each patient by combining clinical data and drug treatments, in order to detect anticholinergic risk and drug-related problems (DRP), such as unnecessary drugs, excess or under-dosing, duplicity, drug-drug interactions (DDIs), potential adverse drug reactions (ADRs), and potentially inappropriate medication (PIM), which was defined according to the last version of the STOPP/START criteria [24].

Severe and moderate DDIs analysis were also assessed with two drug compendia software: Drug-Reax (IBM Micromedex 2022, Greenwood Village, CO, USA) and BOT PLUS database (v. 2021, Spanish College of Pharmacists, Madrid, Spain). Both tools met the minimum quality criteria established by Rodriguez-Terol et al. [25].

The necessary minimum sample size was estimated to be 171 residents, assuming a precision of 0.075, an estimated probability of 0.5 and a significance level of 0.5 [26]. The NH was chosen as it exceeds the minimum sample size, to obtain better precision.

### 2.1. Statistical Analysis

Data analysis was performed with the statistical package SPSS Statistics 26 (IBM Corporation, Armonk, NY, USA). Descriptive statistics (frequencies, median, standard deviations, and ranges) were used to characterize the study population. Logistic regression was performed to identify those variables associated with polypharmacy. Odds ratio (OR) were calculated with their respective 95% confidence intervals (95% CI). Multivariable ordinal logistic regression analysis was conducted to assess the impact of each predictor on DDIs. A *p*-value of <0.05 was considered significant.

### 2.2. Ethical Considerations

The study was approved by the Institutional Review Board of the Nursing Home and the Ethics Committee of the University of Leon (ULE-015-2021) and carried out in accordance with the Declaration of Helsinki.

## 3. Results

A total of 326 residents were screened for eligibility. Those younger than 65 years and with less than 1 month of treatment were excluded. Thus, 222 were selected for the study.

The mean age was 85.5 ± 7.8 years (range 65–107, median 86), and women represented the majority (67.1%). All NH residents showed multimorbidity (two or more chronic conditions). The most common chronic diseases were hypertension (55.5%), cognitive impairment (34.2%) and cataracts (31.5%).

Polypharmacy was present in 78.8% of NH residents, who consumed a total of 1545 drugs, with a median of 7 (range = 0 to 17). Table 1 summarizes the characteristics of the participants (gender, age, number of pathologies, anticholinergic risk, and DRP, including DDIs, ADRs, and PIM) according to the level of polypharmacy. Polypharmacy, excessive polypharmacy and total polypharmacy were significantly associated with increasing anticholinergic risk, the consumption of two or more unnecessary drugs, the occurrence of severe/moderate DDIs, and the existence of PIM. A direct association between excessive polypharmacy and total polypharmacy with seven or fewer drugs involved in potential ADRs was detected, as well as between excessive polypharmacy and two or more duplicities.

The DRPs identified numbered 3111, with a median of 10 DRP/resident. Only in three NH residents (1.4%), no DRP was detected. The characteristics of the different DRPs are presented in Table 2, including the proportion of ATC-N drugs (Nervous System) taking part in DRP as well as those compounds most commonly involved. 

Regarding duplicities (4.3%), we detected 23 (34.3%) with benzodiazepines, four (6.0%) for calcium channel blockers, and three with laxatives (4.5%) and antipsychotics (4.5%).

We also identified a total of 1420 DDIs in 180 NH residents (81.1%). The median DDIs per participant was 4, ranging from 0 to 33. According to CheckTheMeds, 26 DDIs (1.8%) were classified as severe and 325 (22.9%) as moderate. Regarding these severe/moderate DDIs, the median was 1 DDIs/participant (range, 0–13), and they were present in 55.4% of NH residents. Severe/moderate DDIs distribution was as follows: one potential interaction in 48 NH residents (21.6%), two in 27 NH residents (12.2%), and more than two potential DDIs in 48 NH residents (21.6%). Moreover, the number of DDIs was significantly associated with polypharmacy and excessive polypharmacy (Table 1). 

ATC-N group drugs (Nervous System) were related to 97.2% of those severe/moderate interactions (Table 2). In fact, the most frequent drugs involved in these DDIs were lorazepam (17.4%), trazodone (15.4%), mirtazapine (11.7%), escitalopram (11.4%) and alprazolam (9.7%). ATC-N group drugs were also involved in 38.5% of ADRs, and 33.8% of those drugs were classified as unnecessary.

Table 3 shows the most common potential DDIs found, the drugs implicated and their consequences, as well as their severity according to CheckTheMeds software. Almost half of DDIs caused central nervous system depression and nearly one-fifth of QT prolongation. 

As shown in Table 4, benzodiazepines are the drugs most frequently found in potential harmful DDIs, especially with opioids, with a severity ranging from major to moderate, depending on the database considered. Most of them were of moderate severity, according to CheckTheMeds and BOT PLUS, whereas severity was always major in all of those DDIs detected by Drug-Reax.

As for adverse drug reactions (ADRs), we identified 684 drugs involved in potential ADRs, ranging in residents from 0 to 29 drugs. According to CheckTheMeds, 101 NH residents used 1–4 drugs that may trigger ADRs (45.5%), 26 NH residents (11.7%) consumed 5–7 drugs, and 59 NH residents (26.6%) more than seven drugs that may favor the appearance of adverse effects. Proton pump inhibitors (PPI) were the group most implicated in these potential ADRs (89), followed by benzodiazepines (78) and antidepressants (78), and diuretics (68). In this case, multivariate logistic regression revealed that 4–5 (OR: 28.57; 95% CI: 5.18–166.7) or 6–10 pathologies (OR: 12.66; 95% CI: 0.47–10.99) increased 28.5 and 12.7 times the risk of potential ADRs.

Regarding potentially inappropriate medications (PIM), they were not detected in only seven NH residents (3.2%), showing a median of 5 PIM/resident. In more than a half (119 NH residents; 53.6%) five or more PIM were present. Moreover, polypharmacy was strongly associated with an increasing number of PIM, except in the group of excessive polypharmacy in which it was associated only when 5–7 PIM were present (Table 1). The logistic regression did not reveal significant differences between PIM and the rest of the characteristics (gender, age, number of pathologies and anticholinergic risk).

Table 5 displays the ordinal logistic regression analysis performed. DDIs risk was significantly higher with increasing polypharmacy and anticholinergic risk.

## 4. Discussion

Evaluation of polypharmacy is of concern in the elderly, and an important focus of integrated care. The present study has assessed the appearance of DDIs and other potential drug-related problems (DRP) in a certain sample of aged people. It should be noted that the sample employed in this study were NH residents in the province of Leon, which is one of the oldest Spanish regions. So, it would adequately reflect the potential DRP in this country. Furthermore, to our knowledge, this is the first study in which the characteristics of DRP in a Spanish NH have been assessed.

The study confirms a high rate of polypharmacy in the NH residents evaluated, which is in line with other studies [20,21,22]. Prevalence of polypharmacy has been estimated by the WHO in 38.1–91.2% of long-term care facilities [5,27]. The main challenge associated with polypharmacy is the appearance of different DRPs, especially DDIs and ADRs. In the present study, we found a median of 10 DRPs/resident, as well as a high percentage of unnecessary drugs (30.6%), which may trigger potential harmful ADRs (44.3%) and severe/moderate interactions (22.7%). Our study shows a strong correlation between polypharmacy, multimorbidity and the existence of certain DRPs.

Although our frequency of unnecessary drugs (30.6%) is lower than those provided by Fog et al. in 2017 (43.5%) [28] and 2020 (31.9%) [29], it is still very high. Unlike these authors, for which benzodiazepines were the drugs most detected as unnecessary [28,29], in our study omeprazole was the compound most frequently involved. Recent studies indicated that proton pump inhibitors (PPI) were frequently found among the most overprescribed all over the world [30,31], and 30–50% of prescriptions would be inappropriate [32], increasing up to 79.7% in long-term facilities users [33]. Thus, the application of inappropriate medication criteria is strongly recommended for the elderly. In fact, the STOPP/START criteria recommend discontinuing or dose reducing PPI in the elderly with treatments longer than 8 weeks for uncomplicated disorders [24] due to their potential ADRs.

Our findings of excessive dosing (2.5%) and underdosing (1.7%) were much lower than those reported by Fog et al., with values of 12.5 and 2.7% [28] and 14.2 and 3.3% [29], respectively. In our study, escitalopram was the most overdosed (15 or 20 mg/day). For this drug, the FDA in 2012 [34] and the EMA in 2014 [35] restricted the maximum daily dose to 10 mg for adults over 65 years. Citalopram, escitalopram and other antidepressants have also been associated with significant increases in QT in adults of a wide range of ages [36].

Duplicities were detected in 43 residents (19.4%), increasing the risk of ADRs and DDIs. Other authors reported lower values (12%) [37]. Benzodiazepines participated in more than one third of them (34.3%), which may favour sedation, falls and fractures, or mental confusion [38]. Thus, if the use of benzodiazepines is essential, they should be prescribed for the shortest possible time and with the lowest effective dose. On the other hand, drug duplicity implies a higher risk of pharmacological interactions. In our case, most of these DDIs due to duplicities were moderate (50.7%) and severe (10.4%). In a study carried out on outpatient elderly in which the STOPP/START criteria were applied, the most frequent STOPP criterion was duplicity [39], and the same happened in another one developed in a NH in Macau [40]. 

Our NH residents showed a large number of DDIs, although most of them were mild. As described previously, the frequency of severe/moderate DDIs was 24.7%. Reported frequencies of DDIs vary between 15 and 70% [41,42,43,44,45]; 55.4% of our participants were exposed to one or more severe/moderate DDIs, which is higher than the values provided from NHs by other authors (25.5% [11] and 37.8% [40]). As in other studies, a strong association between an increasing number of drugs and DDIs has been observed [46,47], but it should be taken into account that there are discrepancies in the literature due to the different sensitivity of the tools used to detect these interactions [25,48,49,50,51,52]. We chose CheckTheMeds because it is a healthcare informatics tool that helps to screen the treatments of each patient individually and it is commonly used in hospitals; BOT PLUS as it is the database of the Spanish College of Pharmacists, and it is widely employed in this country, and Drug-Reax is also widely used internationally by healthcare professionals. In CheckTheMeds, DDIs were classified into severe interactions (to avoid); moderate interactions (to be monitored), and mild interactions (to know and assess). In the Spanish database BOT PLUS DDIs are categorised into major (with serious clinical significance), moderate (with moderate clinical significance), and minor (with minimal clinical significance). Finally, Drug-Reax groups DDIs are categorised into major, moderate and mild, with different degrees of scientific evidence.

Regarding these discrepancies, the most frequent interactions detected with CheckTheMeds were not always identified by the other two tools. As reported in Table 4, the most common interaction (benzodiazepine + opioid) is listed as moderate in BOT PLUS, but as major in Drug-Reax. Benzodiazepines may exacerbate opioid-mediated respiratory depression, and increase the risk of fractures, visits to the emergency rooms and overdose deaths [53,54]. On the other hand, the second one (benzodiazepine + trazodone) did appear in the Drug-Reax tool and not in BOT PLUS, and severity is described differently in both databases (major/moderate). Other interactions, such as benzodiazepine + venlafaxine and omeprazole + gliclazide combinations, were not detected by any of the other two databases (BOT PLUS and Drug-Reax), and in both cases we only found one reference to support them. In the first case (benzodiazepine + venlafaxine), caution is recommended with this association because it may increase the incidence and severity of ADRs [55]. In the second one, omeprazole may augment the risk of hypoglycemia associated with gliclazide in most patients, as the oral antidiabetic is metabolised by CYP2C19, and omeprazole is an inhibitor of this enzyme [56]. Finally, the interaction known as the triple whammy (angiotensin converting enzyme inhibitors/angiotensin-II receptor blockers (ACEI/ARBs) + diuretic + nonsteroidal anti-inflammatory drugs (NSAIDs)) was detected by CheckTheMeds, but not by the other two databases, which worked only with pairs of drugs. The triple whammy was described by Thomas in the year 2000 [57] and, since then, several studies have reported a decrease in the glomerular filtration rate and the consequent acute kidney injury [58,59]. In this case, the risk of acute kidney injury increases when at least two of these medicines are prescribed together, becoming much greater if all three are combined.

Inappropriate polypharmacy is a worldwide problem in the elderly, as it increases drug costs and the use of health care systems and diminishes their quality of life. In the present study, the prevalence of inappropriate medication was 96.8%, which is much higher than the range reported elsewhere (11.5–62.5%) [60,61]. Comorbidity and polypharmacy increased the risk of ADRs, as in other studies [62]. Prevalence of ADRs in the elderly has been estimated at 11–22% [63,64]. In our study, 44.3% of drugs consumed may trigger potential ADRs, and more than a third of drugs from the N-group (Nervous System) were implicated.

In the last years, improving drug prescription in the elderly has received increasing attention. For this reason, the Spanish Society of Primary Care Pharmacists (SEFAP), based on Milton et al. [65], recommends reviewing medication every 6 months for NH residents with polypharmacy, and at least once a year for the rest of institutionalised people. Furthermore, other authors concluded that it is necessary to integrate the pharmacist into NH in interdisciplinary collaboration with doctors and nurses to identify, solve and prevent DRP [66].

The present study is not without limitations. The relatively small sample size and the assessment of information from a single NH may affect the generalizability of the results, but as we have already explained, Leon is one of the oldest regions in Spain, a country with one of the highest rates of aging worldwide [67]. In addition, we have not considered OTCs, dietary supplements, or herbal medications that may be consumed by the NH residents and may have also accounted for a higher level of polypharmacy as well as the high number of interactions found. Moreover, we should bear in mind that there may be differences between regions and countries when prescribing. Finally, it is a retrospective study; therefore, information was limited to that taken from the medical charts and, on occasions, it was not possible to obtain more information about patient conditions or their pharmacological history before the institutionalization in the NH.

Identification of DRP in NH residents can help to define better prevention strategies to enhance the quality of life of this group of population. Although our findings require further research, may serve to develop guideline strategies. Establishing a detailed understanding of the patterns and characteristics of potential inappropriate polypharmacy in the elderly may provide a basis for minimising the health and economic consequences caused by inappropriate polypharmacy.

## 5. Conclusions

A high prevalence of polypharmacy and DRP in the NH population assessed has been observed and, more specifically, DDIs. Anticholinergic risk and comorbidities are factors significantly associated with polypharmacy. A direct correlation between polypharmacy and anticholinergic risk with DDIs has been established and may trigger ADRs. Moreover, potentially inappropriate polypharmacy also exists among participants. The consequences of the most commonly observed DDIs consisted of central nervous system depression and QT prolongation. The study also emphasizes the need for a regular evaluation of drug prescribing in NH residents to minimize the risk of DRP, clarify those diagnoses that do not need pharmacological therapy and distinguish between appropriate and inappropriate polypharmacy.

## Figures and Tables

**Table 1:** Factors associated with polypharmacy among residents in the NH studied (reference category: non-polypharmacy).

Characteristic	Polypharmacy (5–9 Drugs)	Excessive Polypharmacy (≥10 Drugs)	Total Polypharmacy (≥5 Drugs)
	Odds Ratio (95% CI)		
Gender Male	0.79 (0.39–1.61)	1.47 (0.63–3.44)	0.94 (0.47–1.85)
Age			
76–85 years	1.28 (0.44–3.72)	5.13 (0.94–28.18)	1.71 (0.61–4.83)
86–95 years	1.19 (0.43–3.29)	2.67 (0.49–14.61)	1.35 (0.50–3.67)
≥96 years	1.53 (0.37–6.35)	3.50 (0.43–28.45)	1.75 (0.44–7.04)
Pathologies			
3–5 pathologies	0.05 (0.01–0.22) *	-	0.03 (0.01–0.13) *
6–10 pathologies	0.20 (0.06–0.69) *	0.03 (0.01–0.19) *	0.13 (0.04–0.45) *
≥11 pathologies	0.71 (0.22–2.36)	0.35 (0.10–1.23)	0.57 (0.18–1.81)
Anticholinergic risk			
Low	3.06 (1.26–7.42) *	-	2.44 (1.04–5.72) *
High	53.17 (6.42–440.25) *	58.67 (5.30–648.95) *	54.27 (6.70–439.57) *
Very high	33.61 (8.64–130.74) *	80.67 (14.90–436.64) *	43.02 (11.44–161.81) *
Drug-related problems (DRP)			
Unnecessary drug			
1 drug	1.42 (0.56–3.59)	1.08 (0.17–6.73)	1.37 (0.56–3.38)
2 drugs	4.63 (1.16–13.67) *	9.75 (1.72–55.37) *	5.31 (1.85–15.23) *
≥3 drugs	15.0 (2.95–76.16) *	29.25 (3.45–247.69) *	16.90 (3.40–84.0) *
Duplicities			
1 duplicity	1.25 (0.39–4.06)	2.63 (0.70–9.81)	1.53 (0.50–4.74)
≥2 duplicities	3.47 (0.43–28.22)	13.5 (1.62–112.54) *	5.52 (0.72–42.57)
Severe/moderate DDIs			
1 severe/moderate DDIs	2.92 (1.11–7.70) *	17.5 (4.21–72.76) *	3.83 (1.48–9.89) *
≥2 severe/moderate DDIs	3.21 (1.22–8.42) *	32.08 (8.13–126.63) *	5.01 (1.96–12.81) *
Drug involved in potential ADRs			
1–4 drugs	0.03 (0.01–0.14) *	0.03 (0.1–0.15) *	0.03 (0.01–0.13) *
5–7 drugs	0.32 (0.09–1.17)	0.08 (0.02–0.30) *	0.22 (0.06–0.77) *
>7 drugs	1.03 (0.16–6.70)	0.13 (0.01–1.25)	18.67 (0.10–4.10)
PIM			
1–4 PIM	8.92 (1.03–77.15) *	-	9.57 (1.11–82.67) *
5–7 PIM	98.0 (8.74–1098.5) *	0.01 (0.001–0.05) *	134.0 (12.01–1495.5) *
>7 PIM	156.0 (8.49–2865.04) *	0.27 (0.03–2.85)	288.0 (15.87–5228.01) *

* significant differences (*p* ≤ 0.05). ADRs: adverse drug reactions; DDIs: drug-drug interactions; PIM: potentially inappropriate medications.

**Table 2:** Categories of drug-related problems (DRP), drugs from N-group (ATC classification), and the two drugs most commonly involved.

Drug-Related Problems (n of Drugs = 1545)		Drug Group		Drugs Most Commonly Involved in DRP Listed			
Categories of DRP	n (%)	ATC-N Drugs n (%)	All Other Drugs n (%)	No. 1	n of Drugs	No. 2	n of Drugs
Unnecessary drug	473 (30.6)	160 (33.8)	310 (66.2)	Omeprazole	48	Lorazepam	25
Excess dosing	39 (2.5)	18 (46.2)	21 (53.8)	Escitalopram	11	Digoxin	6
Under-dosing	26 (1.7)	4 (15.4)	22 (84.6)	Nimodipine	6	B3 vitamin	3
Duplicity	67 (4.3)	34 (50.7)	33 (49.3)	Lormetazepam	8	Bromazepam	5
Severe/moderate DDIs	351 (22.7)	341 (97.2)	10 (2.8)	Lorazepam	61	Trazodone	54
Drugs involved in potential ADRs	684 (44.3)	263 (38.5)	421 (61.5)	Omeprazole	64	Furosemide	56

**Table 3:** Drug-drug interactions (DDIs) in NH residents according CheckTheMeds software ^a^.

Potential Outcome	Number of DDIs	Severity of DDIs and Pairs of Drugs Involved
Central nervous system depression	167 (47.6%)	Moderate: Benzodiazepine + trazodone (13); Benzodiazepine + mirtazapine (13); Benzodiazepine + venlafaxine (8)
QT prolongation	11 + 53 = 64 (18.2%)	Severe: SSRI + quetiapine (5) Moderate: SSRI + trazodone (10); SSRI + mirtazapine (8)
Respiratory depression	22 (6.3%)	Moderate: Benzodiazepine + opioid (21)
CYP enzyme inhibitor	9 (2.6%)	Moderate: Omeprazole + citalopram (4); Omeprazole + gliclazide (3)
Serotonin syndrome	3 + 5 = 8 (2.3%)	Severe: Rasagiline + antidepressants (2) Moderate: Escitalopram + amitriptyline (2)
Increased bleeding risk	6 (1.7%)	Moderate: Allopurinol + acenocoumarol (4); NSAIDs + acenocoumarol (2)
Increased adverse reactions NSAIDs	5 (1.4%)	Moderate: corticoids/SSRI + NSAIDs (5)
Triple whammy	3 (0.9%)	Moderate: ACEI/ARBs + diuretic + NSAIDs (3)
Anticholinergic risk	1 + 1 = 2 (0.6%)	Severe: Tolterodine + ipratropium (1) Moderate: Tolterodine + baclofen (1)
Other	65 (18.5%)	
Severe DDIs	26 (7.4%)	
Moderate DDIs	325 (92.6%)	
Total DDIs	351 (100%)	

^a^ In some rows only the most frequent DDIs are shown.

**Table 4:** Most common potential harmful drug-drug interactions (DDIs) and severity classification according to the different tools used.

Rank	Potential DDI	CheckTheMeds	BotPlus		Drug-Reax	
		Severity	Severity	Scientific Evidence	Severity	Scientific Evidence
1st	Benzodiazepine + opioid (21)	Moderate	Moderate	Ample	Major	Fair
2nd	Benzodiazepine + trazodone (13)	Moderate	-	-	Major	Fair
	Benzodiazepine + mirtazapine (13)	Moderate	Moderate	Ample	Major	Fair
3rd	SSRI + trazodone (10)	Moderate	-	-	Major	Fair
4th	Benzodiazepine + venlafaxine (8)	Moderate	-	-	-	-
	SSRI + mirtazapine (8)	Moderate	Major	Isolated	Major	Fair
5th	SSRI + quetiapine (5)	Major	Moderate	Isolated	Major	Fair
6th	Omeprazole + citalopram (4)	Moderate	Minor	Theoretical	Major	Fair
	Allopurinol + acenocoumarol (4)	Moderate	Moderate	Isolated	Major	Good
7th	Omeprazole + gliclazide (3)	Moderate	-	-	-	-
	ACEI/ARBs + diuretic + NSAIDs (3)	Moderate	-	-	-	-

**Table 5:** Ordinal logistic regression analysis showing the association between predictor variables and DDIs (reference category: non-DDIs).

Predictor Variables for DDIs	OR (95% CI)
Low anticholinergic risk	2.96 (2.17–4.06) *
High anticholinergic risk	8.79 (6.42–12.02) *
Very high anticholinergic risk	26.04 (19.03–35.63) *
Polypharmacy (5–9 drugs)	1.78 (1.08–2.93) *
Excessive polypharmacy (≥10 drugs)	3.15 (1.91–5.21) *

* significant differences (*p* ≤ 0.05).

## Data Availability

Data are available on request from the corresponding author.
